# Air and Ventilation
*Library of Illustrated Standard Scientific Works. Chemical Technology. Vol. i, Part i, Fuel and Its Applications. By Drs. Ronalds and T. Richardson. London: Baillière. 1856.On the Defective Ventilation of Hospitals. By John Roberton, Surgeon. Manchester: 1856.Observations on the Construction and Ventilation of Hospitals for the Sick. By John C. Steele, M.D., Superintendent of Guy's Hospital. Glasgow: 1856.Medical History of the Niger Expedition. By J. O. McWilliam, M.D., Senior Medical Officer of the Expedition. London: Churchill. 1853.On the Movement of Atmospheric Air in Tubes. By W. D. Chowne, M.D. London: 1855.On the Smokeless Fireplace, Chimney Valves, and Other Means for Obtaining Healthful Warmth and Ventilation. By Neil Arnott, M.D., F.R.S., F.G.S. London: Longmans. 1856.Memorandum on Asiatic Cholera and Other Epidemics as Influenced by Atmospheric Impurity. By Neil Arnott, M.D., F.R.S. Report to Board of Health. 1854.On the Ventilation of Lighting Lamps. By Professor Faraday. Paper read before the Institute of Civil Engineers, June 27th, 1843.Aération et Ventilation des Hôpitaux, Prisons, et Habitations particuliers. Rapports Généraux des Travaux du Conseil de Salubrité. Paris: 1855.Statical Essays, containing Hæmostaticks. By Stephen Hales, B.D., F.R.S. 2 vols. London: 1733.The Physiological Phenomena of Mountain Sickness. By Stanhope T. Speer, M.D. London: Richards. 1853.On Ventilation, and the Means of Determining its Amount. Lecture delivered before the Royal Institution. By H. Bence Jones, M.D., F.R.S. Reported in the *Chemist* for June, 1856.Effects of Air on Human Bodies. By J. Arbuthnot, M.D. London: 1733.Medical Works of Richard Mead, M.D. London: 1762.


**Published:** 1856-10

**Authors:** 


### JOURNAL OF PUBLIC HEALTH.

---

OCTOBER 1856.

---

### AIR AND VENTILATION.*

In reply to various communications and inquiries, we have from time to time supplied in the Journal of Public Health an epitome of various plans and suggestions, from different authors, bearing on the subject of ventilation. Such notes are, however, too scattered and general to be of direct service to the sanitarian inquirer; and we have therefore thought it would be a practical task, worthy some amount of labour, to review at length the subject of air and ventilation, and to place before the reader a paper on these points,

---

* Library of Illustrated Standard Scientific Works. Chemical Technology. Vol. i, Part i, Fuel and Its Applications. By Drs. Ronalds and T. Richardson. London: Baillière. 1856.

On the Defective Ventilation of Hospitals. By John Roberton, Surgeon. Manchester: 1856.

Observations on the Construction and Ventilation of Hospitals for the Sick. By John C. Steele, M.D., Superintendent of Guy's Hospital. Glasgow: 1856.

Medical History of the Niger Expedition. By J. O. McWilliam, M.D., Senior Medical Officer of the Expedition. London: Churchill. 1853.

On the Movement of Atmospheric Air in Tubes. By W. D. Chowne, M.D. London: 1855.

On the Smokeless Fireplace, Chimney Valves, and Other Means for Obtaining Healthful Warmth and Ventilation. By Neil Arnott, M.D., F.R.S., F.G.S. London: Longmans. 1856.

Memorandum on Asiatic Cholera and Other Epidemics as Influenced by Atmospheric Impurity. By Neil Arnott, M.D., F.R.S. Report to Board of Health. 1854.

On the Ventilation of Lighting Lamps. By Professor Faraday. Paper read before the Institute of Civil Engineers, June 27th, 1843.

Aëration et Ventilation des Hôpitaux, Prisons, et Habitations particuliers. Rapports Géneraux des Travaux du Conseil de Salubrité. Paris: 1855.

Statical Essays, containing Hæmostaticks. By Stephen Hales, B.D., F.R.S. 2 vols. London: 1733.

The Physiological Phenomena of Mountain Sickness. By Stanhope T. Speer, M.D. London: Richards. 1853.

On Ventilation, and the Means of Determining its Amount. Lecture delivered before the Royal Institution. By H. Bence Jones, M.D., F.R.S. Reported in the *Chemist* for June, 1856.

Effects of Air on Human Bodies. By J. Arbuthnot, M.D. London: 1733.

Medical Works of Richard Mead, M.D. London: 1762.

to which he may afterwards turn, as to a literary resting-place, where may be found the histories of a few facts, and the index to more.

Dr. Arnott, in his excellent work on *The Smokeless Fire-place and Ventilation*, has made some admirable observations on the comparatively modern application of the principle of ventilation in public and private buildings. He shews that, a century or two ago, men were not even aware that the atmosphere was a "thing;" and that they have only become acquainted with this fact, as they have been taught that gases can be measured like water, and have learned some of the properties of the element oxygen, which the immortal Priestly discovered in the latter part of last century. Hence one of the essentials of life, a sufficiency of pure air, has been considered of little account.

It is to be regretted that, even now, although oxygen has been discovered, and gases have been measured like water, and Dr. Arnott has written a philosophy in simple style, no due importance is paid to the matter in hand by the public at large. Nay, it is further subject of regret that the medical profession as a body, advanced as it is, is not yet alive to the full extent, end, and meaning of efficient ventilation in its applications to the promotion of health and the treatment of disease. Old traditions, fancies, and fables linger yet around the Esculapian priest; still the alarm about chills and draughts perplexes; and still the danger to the patient of leaving his bed or his room in days of returning health, " lest a relapse be the consequence", is presented to the mind as a scarecrow, which need only be walked up to in a cool humour to be mercilessly and safely demolished.

The uses of air to man and his lower earthmates are not easily comprehended at first sight; they are so varied, and, to the uninitiated, so subtle. The effect of the ordinary atmospheric pressure on the body is a first essential to healthy life. It is by this pressure, exerted uniformly over every point of the body, that the corporeal framework is the more firmly knit together. We are, as it were, casts in moulds of air. Removed to a mountain height, where the atmosphere is lighter and the pressure diminished, the body suffers, to borrow a description from Dr. Speer, " with symptoms (varying in degree and number in different individuals) of vertigo, headache, dyspnœa, constriction of the chest, palpitation, syncopal tendency, oozing of blood from the mucous surfaces, increased rapidity of the pulse, nausea and vomiting, thirst, febrile tongue, muscular pain, sense of extreme debility in the lower limbs, and general prostration of strength" —results arising, according to the same author, from a threefold source: congestions of the deeper portions of the circulatory apparatus, increased "venosity", in plainer words, impurity of the blood, and a loss of equilibrium between the pressure of the external air and that of the gases existing within the intestines.

The symptoms thus induced bear, in the opinion of Dr. Speer, a close analogy to those of an ephemeral fever: a remark of great importance in a physiological point of view.

Similar symptoms may be produced in animals, by the removal of a portion of the atmosphere from a vessel in which they are confined, and in other ways. If, for instance, to the ordinary pressure of the atmosphere on the body some general mechanical pressure be added, such as the application of a moderately tight bandage to a limb, the artificial compression is tolerably well borne when evenly applied. But if such bandage be now rapidly removed, the relief is painful, the blood rushes into the recently contracted vessels, the skin becomes red, and a painful tingling sensation is, for a moment or two, experienced. If this artificial pressure be extended to the whole body, these results are more striking. They represent, in a minor degree, the effect of removing the atmospheric pressure. Note the following:

The holy Fathers of the Inquisition, of blessed memory, in the gentleness of their natures, and with surpassing ingenuity, had an interesting physiological experiment of this nature for chastening the erring sons and daughters—specially the daughters—of Eve. To wit, they swathed the bodies of their sinners very firmly from foot to shoulder, and when this pressure was just tolerable, they suddenly removed it. The result supplied the soul-healing chastisement. The pain of relief was exquisite; the prostration decided; while the febrile warmth which succeeded gave an admonishing foretaste of what all sinners might ultimately hope for who should offend the inquisitorial and priestly will.*

But to return: there is another use to which the ordinary atmospheric pressure extends. Under its influence the lungs are kept charged with air. When the chest is expanded in the act of inspiration, the pressure of the atmosphere causes the lungs to fill with air, and to follow up the expansion of the walls of the chest. During the act of expiration, on the other side, the entrance of external air into the lungs

* See Arbuthnot, On the Influence of Air on Animal Bodies, p. 43.

is, in a great measure, prevented for the moment, and the external air itself becomes the recipient of the expired gases.

Thus, the mechanical effects of atmospheric pressure on the body are most important, and the effects of extreme variations of pressure are well mapped out by certain striking symptoms. How far the effects of those lesser variations of pressure which occur at ordinary levels, and which are indicated by ordinary barometrical changes—how far these exert a physical influence on the body, is as yet a question open to an extended series of observations and experiments.

A second important use of the atmospheric air relates to its influence on the animal temperature in a physical point of view. As the heat made in the body passes off from it at every point by radiation, it commingles, to use a common term, with the atmospheric medium in which the body is encased. Thus, if the external atmosphere be cooled far below the level of the body, then the radiation of the animal heat is so vigorous, that an arrest of vitality, more or less marked, according to the degree of cold, becomes necessarily manifest. A great variety of physiological changes in this case supervenes. Nutritive acts, which are dependent for their continuance on a full development of caloric, are impeded. The great chemical changes of respiration, the absorption of oxygen, and the evolution of carbonic acid, are reduced; internal congestions follow, and the circulation fails in power; lastly, the soft structures, losing their expanding principle, shrink, the capillaries contract, merely in all probability from the loss of caloric: and the whole of what in the aggregate sense may be called the vital forces are, in a greater or lesser degree, checked in their course.

On the opposite side, when the temperature of the external air is raised towards a level or above that of the body, the due radiation of heat is checked; then the reverse of the above conditions obtains. The nutritive and destructive changes of tissues are accelerated; the muscles are relaxed; the capillaries easily dilate; and the secretions are profuse, from the skin, the lungs, and the kidneys. But this exalted condition of body can no more be tolerated than the opposite, for any length of time. The chemical forces of life are here too actively engaged; they must be brought back to steadier play, or they will stop altogether.

The happy medium for equalising the temperature of the body is the atmosphere. We instinctively perform something for ourselves in this respect, in the use of clothes, of which we put on more or less as sensation tells us. But it is our air garment, or mould, which does most. If this naturally retains an equality, its 60° to 70° Fahr., we are comfortable; if it, capricious, dance about from 32° or less up to 90° or more, we must put it under some certain rules,—must confine it in sections, in rooms where we live, and artificially warm it in the first case; must give it free vent, and lower its temperature by agitation, in the second case. Upon a due consideration of these facts the science of warming and ventilating rooms and buildings essentially depends; as does also the manner of clothing the body, so as best to enable it to meet the vicissitudes in the temperature of the enveloping air.

But the atmosphere does yet more in the way of aiding and abetting the body to live. It removes from the body its volatile refuse, and distributes this refuse into space.

Dirty individual living in St. Giles's may eschew water, and go to his grave with the solid refuse of his *corpus insanum* sticking to him so closely, that dust is added to dust in more senses than one; said individual has not, however, the same power of retention over his own volatile refuse, which is too bad—at all events in a free country. For the inexorable atmosphere, blowing at him wherever he goes, will have its dues from him in shape of carbonic acid, water, ammonia, and what not—will have them, we say, will sweep him clean of them, as far as it is possible. Twenty cubic inches of impure air from the lungs at every expiration must needs be yielded; about as much, says Dr. Arnott symbolically, as would make up the bulk of a full-sized orange, if it could be seen—which is not the case, all for a wise purpose. For if dirty individual, street-crossing sweeper perchance, could see himself thus blowing off an orange, or anything like it, some twenty times a minute, and gulping in another as often, he would inevitably think the task an imposition, and shirk some part of it, as soon as his first alarm was over. Therefore, for obvious and cogent reasons, the work is invisibly, but not less surely, done; there is an escape from the body of the impure vapour already described, while a supply of pure vapour, oxygen and nitrogen and water, equal altogether to the loss implied, is taken in.

But let us follow (keeping to Dr. Arnott's peculiar simile) the "orange" as it is evolved. Having left the chest and got out of doors, it rises just as a soap-bubble would, or a balloon; for being itself at the moment of expulsion heated to nearly the temperature of the body, viz., 96 degrees Fahr., it is of lighter density than the atmosphere, floats up in it, becomes diffused, and is at last, by wide spreading, brought to nought.

Dirty individual aforesaid may, if he will, "think of this when he smokes tobacco." There is something more philosophical in smoking a pipe than the vulgar are aware of; for, as the curling fumes ascend from the lips and evanish in mid air, do they not afford to the learned eye a clear view of the "orange", do they not paint the breath sky blue, and shew better than all Dr. Arnott's diagrams of oil and water, and tubes and valves, that air is a "thing"? We ask pardon of the Anti-tobacco Society for this absurd digression.

As, then, the heated vapour from the breath escapes, it rises, and a purer proportion is inspired. What becomes of the invisible "orange" thus thrown off, and why it does not come back, in part at least, direct on the head of the individual, is due to the principle of "diffusion", on which a word must be said.

When two liquids of different densities are put together—say oil and water—they do not remain long in the happiness of a united pair. The oil, taking advantage of the density of the water, floats to the top, and makes for itself a very comfortable water-bed, where it reclines uncontaminated, as all may see. But if two gases of different densities, such as carbonic acid, a heavy gas, and hydrogen, a light gas, be mixed together, they continue mixed, as free gases be it observed, but commingled freely; nay, if a jar of light gas be inverted over a jar of heavy gas, so that the two gases are brought thus simply into communication, the two at once agree to unite in the most friendly fashion. The heavy gas politely rises, the light gas gallantly descends, and in the end there is a complete admixture—brandy and water are not more accommodating to each other.

The discovery of this tendency on the part of one gas to diffuse into another gas was made by Dalton, sandal-maker to the immortals, and himself immortal cobbler, who pushed his argument by demonstration so far, as to lay down a kind of axiom to the effect, that gases of different kinds and densities afford no resistance to each other, but run into each other as they would into a vacuum; and although the labours of Professor Graham have shewn that the diffusion process takes place in different gases with different degrees of rapidity, and that gases are not absolutely vacua to each other, yet the final result is the same as though they were; since it has been proved that gases do, in fact, rush into a vacuum with velocities corresponding to the numbers which have been found to express their diffusion volumes, *i. e.*, with velocities inversely proportional to the square root of the densities of the gases.

Of the cause of this mutual diffusion of gases we are not so clear. There is yet another great law to be discovered in this direction. But it is sufficient for our present purpose to know the fact, that no gaseous matter can be set free, whether from the animal body or from aught else, without being at once freely diffused in the great ocean of the atmosphere, as though practically it were being spread through a great vacuum.

It is impossible to overestimate the magnificence of meaning implied in this grand natural law. This great aerial sea, forty-five miles deep each way from any point of the earth's surface, and into which we insignificants can but raise ourselves with the help of bricks and mortar some few poor hundred feet; nay, into which our madcaps who try to pierce heaven on a wind-bag cannot penetrate more than a mile or so; this great aerial sea, how competent for the end had in view, how capacious a chamber for the distribution of coal smoke, human breath, volcano vapours, and poisons innumerable, from soothing nicotina out of dirty individual's lips in St. Giles's, to concentration of irrespirables from the top pot of the St. Rollox, far north!

And, lastly, the winds must not be forgotten. Think of winds—great agitations of great atmosphere in the great chamber, always mixing up everything on the large scale, as if some enormous Maelström were perpetually at work in the universe; but all in order, all according to the first law—Order. The wind movements, however, and all the effects of admixture springing out of them, must be carefully removed from the diffusion process. That is to say, there must be no relationship of cause and effect introduced between them. None such exists. The diffusion process is a fact *per se*, it takes place in air in a state of rest, the same as when such air is in a state of motion.

There is a fourth, and yet more important use of all, which the air around us plays in regard to the men and animals who live at the bottom of it, as at the bottom of an immense air lake or sea. The scientific world now-a-days recognises air to be a food, as much a food in its way as beef and potatoes in their way. The old saying about the chameleon,

> "Stretched at its ease the beast I viewed,
> And saw it eat the air for food,"

is thus no poet jingler's fancy, but a fact. Air is food; the first food of man and of everything that lives. The chief sustaining element of the air inspired in breathing is the oxygen, which forms a fifth of the whole of the atmospheric sea. The nitrogen, which forms the remainder of the sea, and is usually said to be the mere diluting medium of oxygen, is, however, not altogether inert, for a small portion of it taken in by each inspiration is made use of, or at all events does not return in expiration. The quantity of air received into the lungs of the healthy man at each ordinary inspiration is about twenty cubic inches. This is but a small portion of that which he is capable of receiving, for his chest has a capacity for at least two hundred inches, when well filled, so that he has a reserve portion always in store,—a bank of air. The process of diffusion of gases is here also brought into play; for before the expired air leaves the lungs, the products of waste, which are to be cast off from the blood, are diffused into the air with which the lungs are steadily filled. This provision is admirable, in that it keeps the interchange of pure air for impure air in the lungs going on in one unbroken current, places the command of the respiratory act in a partial degree under the will of the individual, and prevents mishaps which might arise, such as the temporary suspension of respiration, from becoming immediately fatal.

The lungs in health are thus always charged with air; and this air, carried to the extreme ramifications of the air-tubes, and thence into the air-cells, of which there are in the human lungs about six hundred millions, is brought into indirect contact with the blood, which is circulating round the lungs from the right to the left side of the heart. We say indirect contact, because the air-cells are lined with membrane on their part, while the blood which plays over them is itself also enclosed in plexuses of vessels, almost infinitely minute, so that there is an intervening membranous screen between the air and the blood. Here, in this fine but expansive network of air-cells and blood-tubes, do the interchanges of air and blood take place through the membranes. Here the blood returning from all the body, through the right side of the heart, gives off its gaseous refuse; and here the same blood is reinvigorated by the absorption of oxygen and the small quantity of nitrogen already spoken of, with possibly some amount of water. The oxygen, if not the nitrogen, is *food;* and thus the process of respiration is another process of digestion. Going round with the blood to all parts of the body, this oxygen supports all the acts of nutrition; helps to build up the new, assists to remove the old; the phenomena of life are, in fact, all described in the term oxygenation.

Springing out of these considerations, there is yet one other important fact to be considered in relation to air and man: we mean the generation of animal heat. We have seen that the atmosphere is the grand external medium for regulating the temperature of animal bodies; it is also the grand medium for supporting the temperature of these bodies. The process of animal heat-making is again a process of oxygenation, not specially carried on in the lungs, it is to be understood, but in every part of the body where oxygen enters into combination with tissue so as to give rise to a new combination. Of course there is heat liberated, when in the pulmonic system, the oxygen enters into its combination with the blood; but there is also heat produced in other combinations into which the free oxygen absorbed into the blood enters in its round of the general system; *i. e.*, in its unions there with carbon, with sulphur, and with phosphorus.

Thus, to epitomise, we see in the air a variety of uses. It affords mechanical support; it is a heat modifying medium; it swallows all gases exposed to it; it supplies a food to man, out of which he is in part built up; it feeds him with the active principle by which the warmth of his body is sustained.

Upon such simple processes as these, coupled with other processes for the supply of food through digestion, the whole system of organised life depends; and hence some writers have drawn an analogy between animal man and the steam engine, an analogy remarkable in all leading points.

"Allow us", says Dumas, in speaking of the vegetable world, as supplying the necessaries of existence, "allow us to borrow from modern science an image sufficiently grand for comparison with these grand phenomena, and to compare the actual vegetable world, the true magazine from which the animal world derives its elements, with that other magazine of carbon, formed by the ancient deposits of coal, and which, burned by the genius of Papin and Watt, has produced carbonic acid, water, heat, motion, one would almost say life and intelligence."*

Dr. Arnott also gives the following table of comparison:—

---

* Permettez donc, qu'empruntant aux sciences modernes, une image assez grande pour supporter la comparaison avec ces grands phénomènes, nous

| "THE STEAM ENGINE IN ACTION | "THE ANIMAL BODY IN LIFE |
|---|---|
| Takes: | Takes: |
| 1. FUEL, viz.—Coal and wood, both being old or dry vegetable matter, and both combustible. | 1. FOOD, viz.—Recent or fresh vegetable matter and flesh, both being of kindred composition, and both combustible. |
| 2. WATER - - - - - - | 2. DRINK (essentially water). |
| 3. AIR - - - - - - - | 3. BREATH (common air). |
| And produces: | And produces: |
| 4. STEADY BOILING HEAT of 212 degrees by quick combustion. | 4. STEADY ANIMAL HEAT of 98 degrees by slow combustion. |
| 5. SMOKE from the chimney, or air loaded with carbonic acid and vapour. | 5. FOUL BREATH from the windpipe or air loaded with carbonic acid and vapour. |
| 6. ASHES, part of the fuel which does not burn. | 6. ANIMAL REFUSE, part of the food which does not burn. |
| 7. MOTIVE FORCE, of simple alternate push and pull in the piston, which, acting through levers, joints, bands, etc., does work of endless variety. | 7. MOTIVE FORCE, of simple alternate contraction and relaxation in the muscles, which, acting through the levers, joints, tendons, etc., of the limbs, does work of endless variety. |
| 8. A DEFICIENCY OF FUEL, WATER, OR AIR, first disturbs, and then stops the motion. | 8. A DEFICIENCY OF FOOD, DRINK, OR BREATH, first disturbs, and then stops the motion and the life. |
| 9. LOCAL DAMAGE from violence in a machine is repaired by the maker." | 9. LOCAL HURT OR DISEASE in a living body is repaired or cured by the action of internal vital powers." |

We have recorded these general observations on the influence of air on animal bodies, because, in the absence of knowledge bearing on such subjects, it is impossible either to understand the objects of ventilation, or the means to carry out any efficient plan of ventilation. We have shewn that the air is of great service by the pressure it exerts; that it acts as a warmth modifier; that it disposes of gases and vapours; that it supplies nourishment to the body; and that it sustains the animal heat. These things considered, the importance of a free ventilation, of a steady current of pure air around the animal body becomes at once manifest to every mind. As in the steam engine so in the man, no movement, no action can be well done without the free vent of air.

Dirty individual of St. Giles's, removed from street crossing to stoke at a steam engine, polish it up and keep clear its breathing holes, is doing for his inanimate brother the thing he would scorn to do for his animate self. What if the iron horse were to demand a huge pipe and tobacco, and choke up his throat by volumes of smoke? Stoker, smoking his own

---

assimillions la végêtation actuelle, véritable magasin où s'alimente la vie animale, à cet autre magasin de charbon que constituent les anciens dépôts de houille, et qui brûlé par le génie de Papin et de Watt, vient produire aussi de l'acide carbonique, de l'eau, de la chaleur, du mouvement, on dirait presque de la vie et de l'intelligence.

pipe the while, would call his inanimate brother a blockhead, and pull the huge pipe away, as a stupid interference with the due performance of iron horse respiration and activity.

Nomadic tribes, living mainly out of doors, or covering themselves in from the rain and wind in canvass tents, take and require but little care in regard to ventilation, since they interfere not with atmospheric laws. But when men begin to erect stone walls, to hedge themselves in from the atmosphere with impermeable barriers, and to pollute their confined dwellings with their own emanations, the matter is reversed. Supreme nature is defied, and the defiants pay the forfeit always visited on such delinquency. Under these circumstances the principle of ventilation, as it is called, has slowly been evolved out of nothing, and has suggested various ways and means by which the atmosphere may be made subservient to the designs and life methods of men. Even in the early days of architecture, when real stone or wood houses were first built, the necessity for any special attention to ventilation was uncalled for. In these days, when light was admissible into the building by means only of large open casements, the air went in with light, and was as freely diffused. In the old Roman house, with its *compluvium* and numerous unbarricaded windows, the ventilation must have been perfect and simple. But when it became the fashion to let in the light alone, and thus to cut off the air from the same entrances, then the evil effects of confined air in houses became a necessity, in the absence of some new means for its free admission and after removal. This inconvenience was felt the more in places where great numbers of people were placed together in one limited spot; as on board ship, in the wards of a hospital, the compartments of a prison, or even in the closely built streets of a town. What amount of disease, what amount of death, must have ensued for many centuries from absence of pure air, and whilst men were ignorant of the actual value of air in its relation to life and health, it is impossible to say.

For many ages after the days of the old Roman literature, little of worth is met with on the vital properties of air, and the importance of a free air current. For sound instruction on these points, the world had to wait for Dr. Hales, whose physiological labours are amongst the most remarkable records of natural science in the beginning of the last century. Unable, in his day, to make a correct analysis of air, Hales nevertheless succeeded in showing, with remarkable correctness, the physical and even the chemical virtues of this gaseous material. He showed that air was a "thing;" he measured the 220 cubic inches of air which the chest can contain; he measured the number of cubic inches drawn in at each inspiration; he even approximated to the loss sustained in the lungs by the absorption of oxygen and nitrogen; whilst in his researches on the re-respiration of expired air, he describes unconsciously the physiological influence of carbonic acid.

With these facts before him, Hales was not slow to see the importance of free ventilation. "Thus", he observes, "what we call a close warm air, such as has been long confined in a room, without having the vapours in it carried off by communicating with the open air, is apt to give us more or less uneasiness, in proportion to the quantities of vapours which are floating in it. For which reason the German stoves, which heat the air in a room without a free admittance of fresh air to carry off the vapours that are raised, as also the *modern* invention" (A.D. 1733) "to convey heated air into rooms through hot flues, seem not so well contrived to favour a free respiration as our common method of *fires in open chimneys*, which fires are continually carrying a large stream of heated air out of the room up the chimney, which stream must necessarily be supplied with equal quantities of fresh air through the doors and windows, and crannies of them."

But we must follow Hales a step further. "Two gallons of air", he states, "breathed to and fro for two minutes and a-half become unfit for respiration. Whence no wonder", he continues, "that the air should be infected, and apt to breed distempers in close prisons, where not only the breath, but also the plentiful perspiration of many confined together stench the air, and make it apt to breed what are called gaol distempers, which inconvenience might in a great measure be prevented if gaols were so contrived as to have a free passage for the wind to blow through them, and thereby communicate fresh air, for want of which many of those unhappy persons are not only deprived of liberty in gaols, but too often of life also."

Speaking of the ventilation of ships, the same author refers to the old plan of washing the beams and decks with vinegar. This, he says, can never take the place of a thorough ventilating air; but "there may be a ferment between this acid and the then too alkaline air, which may thereby be reduced in some degree from its alkaline to a neutral more wholesome state." Who told Stephen Hales about an alkaline air in crowded, unventilated rooms it is hard to say. We infer that Stephen was a fast philosopher.

Arbuthnot, following Hales, gave some sound views regarding pure air. The respiratory act he designates as "the second digestion", and comments upon the value of a healthy respiration in all disorders.

Sir John Pringle, in his important work on the *Diseases of the Army*, published in 1768, dwells with great emphasis on the importance of a free ventilation in hospitals. His rule for preserving the purity of such places is a sound one, viz., "to admit so few patients into each ward, that anyone unacquainted with the danger of bad air might imagine there was room to take in double or triple the number. I have generally found", he adds, "those rooms most healthful where, by broken windows, and other wants of repair, the air could not be excluded."

In the event of a room being deficient of a chimney, which, when present, acts as a constant ventilator, Sir John recommends the use of Dr. Hales' ventilators, and appends a note written by Dr. Hales on the manner in which these ventilators are to be applied. "A board was to be screwed fast to the upper part of a window on the outside of each room. This board was to have a round hole in it, and also the glass opposite to it, of a size sufficient to receive a trunk of a sufficient length to reach from the window *to a small ventilator* on the ground, through which the foul air was to be drawn out of each room, the fresh air entering in at the door. The same trunk would serve for windows of different heights by being placed more or less obliquely. A very small ventilator will be sufficient for this purpose—about five feet long and twenty inches wide."

The ventilators of Dr. Hales, here noticed, were a novelty in their day. They were not exactly new to the world, for the old Romans had invented a bellows for the ventilation of mines, of which Dr. Hales' machine was merely a modification, and hardly an improvement.

However, in the Hales ventilators, a revival was instituted, which the Royal Society had the privilege of hearing of in 1741.* The atmosphere was to be made now the slave of the builder. A man with mysterious bellows under his arm offered his assistance and even patronage to His veritable Royal Highness the Prince of the Power of the Air. His

* Dr. Hales was also the author of a "Treatise on Ventilators".

Highness was tickled at first, but objected to the interference when he found the man was in earnest.

The mysterious bellows themselves, called by their author in reference to their application to ships, "the ships' lungs", consisted innocently enough of a square box, divided transversely by a horizontal partition of wood. This partition, being supported by two hinges fixed in the centre of one of the ends of the box, admitted of being moved up and down at its other end by means of a rod, which passed through an opening in the upper side, or top of the box. At that end of the box to which the hinges of the centre partition were attached were four holes—two above, and two below the partition. These holes were armed with valves, the two upper ones opening—one inwards, the other outwards, the two lower ones opening also—one inwards, the other outwards. By this arrangement, whenever the partition within was moved up and down at its free end a change of air was established. As the partition went down, the air in the lower compartment was forced out in part through the valve opening outwards, while pure air was admitted into the upper compartment through the opening, the valve of which opened inwards. As the partition went up the action was reversed; but the result was the same. The machine was worked by the hand, and on board several men were employed to pump at intervals the impure air out of the lower parts of the vessel.

Another machine, an improvement on the Hessian bellows, was also invented about the same time by the Rev. Dr. Desaguliers.

The ventilation of Dr. Hales was applied extensively in the navy when first brought into notice; for Hales had influence at head quarters; was patronised by Prince Frederick; became the almoner of the prince's dowager princess; and might have been a bishop if his tastes had run in that high and mighty direction.

The ventilation, however, failed, for reasons which Dr. Arnott thus points out:—"The valve apertures were each only one forty-fourth part as large as the surface of the piston", and "caused expenditure of eighty-eight times the force that would have moved the air if there had been no such impediment. The labour implied in the working of Dr. Hales' machines led to their discontinuance."

Improving on this principle, Dr. Arnott has, in these days, invented a pump, which he calls the "single ventilating pump". Its action is exceedingly simple, and it has been employed effectually on board the *Anson* convict ship.

To go back. Soon after the appearance of Dr. Hales' scheme, a new plan of ventilation was suggested by Mr. Samuel Sutton, which met with the approval of the distinguished Dr. Mead, and was submitted to the consideration of the Admiralty authorities in 1741. We notice this plan specially, because it introduces a systematic attempt to make a chimney a ventilating shaft. Mr. Sutton, in fact, made a discovery, which is here given,—Dr. Arnott being respectfully requested not to laugh until he has read the whole.

(Mr. Sutton *loq.*) "I at length found that by stopping the air out of a room that had three fireplaces, and making two large fires in two of them, I could bring the air to draw down the blind chimney" (syphon ventilation on a grand scale) "with such force as to put out a candle."

Pushing his experiments still further, Mr. Sutton concluded, "that a fire being always kept on board ship, and a pipe or cavity made to the well, one end of it being heated by fire, a change of air would follow, and by this means be rendered sweet and pure and fit for respiration."

In the application of this scheme to the ventilation of ships, Mr. Sutton proposed that tubes from various parts of the ship should be made to open into, or rather beneath, the furnace of the great copper, in which the provisions of the crew were cooked; he thus supplied the fire with air from all parts of the ship, and kept up a constant current throughout all the compartments.

Concerning some interesting difficulties which beset Mr. Sutton in getting his plan tried in his majesty's ships; how he waited by appointment on Sir Jacob Ackworth, surveyor of naval works, at seven in the morning, and waited all day, to get at last a rude and unsatisfactory interview; how he was bandied about from one department to another; how, in a word, he got into the Circumlocution Office of those days, and got out of it again; concerning all these things, and a great many more, let the reader interested in Circumlocution history refer to the work of Dr. Mead.

The primitive ventilation contrivance which it was Mr. Sutton's ambition to annihilate in ships, and to supersede by his own, was, nay *is*, the famous "wind-sails plan". The wind-sails are made of sailcloth, and are usually between twenty-five and thirty feet long, according to the size of the ship. They take the form of a cone ending obtusely. When they are used, they are hoisted by ropes to about two-thirds or more of their height, with their bases distended circularly by hoops, and their apex hanging downwards in the hatchways of the ship. Above each of these one of the common sails is so disposed, that the greatest part of the air rushing against it is directed into the wind-sail and conveyed, as through a funnel, into the lower part of the ship. This mode of ventilation is inefficient, cumbersome, and troublesome, but Mr. Sutton did not succeed in putting it down. It flourishes still, but in some ships metal funnels ending in tubes are now used instead of the wind-sail.

Since the time of Dr. Hales and Mr. Sutton, many other ventilation schemes have been proposed. We must, however, be content to refer only to such in the sequel as are of the most modern date.

The genius of these latter days has bewildered itself and others so extensively in devices of ventilation, that the saying of a wretched punster, "the whole question of ventilation requires to be thoroughly ventilated", conveys some touch of truth. To sum up in a few words what the genius of the age has done in this respect, however, would be but to say that it has inherited all the ideas of its grandfather, which ideas it has profoundly vocabularised, and reduced in some cases to the most difficult formulæ. The primitive ideas are not more than three in number. 1. To ventilate by heat. 2. By a pumping process. 3. By no process at all, except by the pressure and movements of the atmosphere, without let or hindrance; old nomade system modernised. Let us consider these plans in order, and refer first to Dr. Arnott's views of the uses of chimneys, and his own ventilating valve.

"What is incorrectly called the chimney-draught", says Dr. Arnott, "is a force exactly equal to the difference of weight between the dilated air in the flue and an equal column of the external atmosphere. This explanation shows why chimney-draught is directly proportioned, first to the dilatation, and therefore to the heat of the air in the flue, and then also to the length or height of the chimney, and it accounts for many of the common cases of smoky chimneys, and suggests appropriate remedies. An influence often overlooked, but of considerable importance, because it continues in operation during the summer, is produced by wind blowing directly across the end of an open tube, such as a chimney-pot. The stream of air, splitting on the chimney-pot, causes a degree of vacuum at the mouth, towards which the air from below moves."

"In sitting-rooms, bed-rooms, nurseries, and enclosed places generally, where people assemble, the impure air of the breath, the burned air from lights, the odour of dishes, etc., because heated and therefore specifically lighter than common air, all ascend first towards the ceiling; but, as in ordinary rooms, no opening exists there for escape (for an open window-top in a room which has an open fireplace only admits the cold air), they soon contaminate the whole air of the room down to the level of the chimney mouth, through which only can any portion ultimately pass away.....

"The ventilating valve is placed in an opening made from the room into the chimney-flue, near the ceiling, by which all the noxious air above referred to is allowed at once, in obedience to the chimney draught, to pass away, but through which no air or smoke can return. The valve is a metallic flap to close the opening, balanced by a weight on an arm beyond the hinge. The weight may be screwed on its arm to such a distance from the axis, or centre of motion, that it shall exactly counterpoise the flap; but if a little further off it will just preponderate, and keep the flap, when not acted on by entering air, very softly in the closed position. Although, the valve, therefore, be heavy and durable, a breath of air suffices to remove it; which if from the room, opens it, and if from the chimney, closes it; and when no such force interferes, it shuts. The valve is so adjusted originally as to settle always in the closed position. An important part of the arrangement is thc wire, which descends like a bell-wire from a valve to a screw or peg, fixed in the wall within the reach of a person's hand, by acting on which the valve may be either entirely closed, or left free to open in any desired degree. In cold weather, or with few persons in the room, the valve, when opened only a little, allows as much air to pass as is requisite. A flap of thirty-six square inches area is large enough where there is good chimney-draught for a full-sized sitting-room with company.

"It is to be observed that if the opening or throat of the chimney-flue over the fire be so wide that more air can easily enter there than can escape at the chimney-pot above, the chimney will not take air in also at the ventilating valve. It is essential, therefore, that with ordinary grates the register flap be so far closed, that when the fire is lighted, little more than the true smoke shall be allowed to enter; and not also, as is usual, much of the pure air of the room escaping with it to waste. A second great fault in common fire-places is the large space left between the fire and the chimney throat, in rising through which the true smoke contaminates much good air, which must then be allowed to pass away as smoky air."

Such is Dr. Arnott's valve; it has been extensively adopted, and when correctly fitted up, it acts well.

A second process for ventilation by means of the chimney has been patented under the title of the "Syphon Ventilator." We have described this plan in the JOURNAL OF PUBLIC HEATH for June 1855. Essentially it consists in bringing down a tube from the upper part of a room and making it enter the chimney at the lower part, or mouth.

This system has been also applied to lamp burners, a tube being carried from the burner into the chimney, so that a current of air is constantly being draughted from above the burner, through it, and so into the main shaft. Dr. Arnott hits mercilessly hard at this plan, stating that the descending tube weakens the force of the draught. There can be no doubt that the term "syphon ventilator", is unhappy and even incorrect. But for removing the air from gas burners the principle succeeds well, and a room may be temporarily ventilated pretty effectually on this plan, by simply carrying a pipe from the top of the room downwards to the chimney, and bending its lower end so as to make it turn into the chimney shaft.

A third patented process is that of Mr. Watson of Halifax. It consists in splitting the chimney into two parts by means of a diaphragm, or central partition. Under these conditions the smoke from the fire is presumed to ascend up by one of the divisions of the chimney, while a current of air descends downwards through the other division. The same plan has been suggested by Dr. Cowan (see JOURNAL OF PUBLIC HEALTH for December 1855), the idea being taken from the statement of Commander Priest, an officer of the royal navy, who found that by straining a piece of canvass vertically across the deck of a ship at the opening to the engine room, the temperature below was rapidly lowered. Mr. Watson's plan has been adopted in the General Post Office, London, and in many public buildings; but we are not able to record its success.

Various attempts have been made of late to effect a perfect system of warming and ventilating at the same time. In the prison of the Mazas, in Paris, this has been attempted by M. Grouvelle. "The air supplied to the cells in this prison is heated" (we take our description from the work of Drs. T. Richardson and Ronalds), "by contact with pipes containing hot water. Ventilation is produced by a vast chimney, about 40 square feet in section, and nearly 100 feet high, which is situated in the centre of the edifice. The whole of the air from the cells is drawn by the action of this chimney in a downward direction through a vertical pipe in each cell, which being in connexion with a night stool, serves, at the same time, for removing excrementitious matters. The pipes from the several cells terminate in an underground vault, whence the vitiated air is drawn off by the chimney draught. A balcony extends along each corridor, at the height of the first and second stories, on to which the cell doors open. Channels are carried below these balconies, in which two sets of cast iron pipes convey currents of hot water in opposite directions. The channel is intersected by partitions, corresponding with the walls of each cell, and the air from the corridor is admitted to the spaces between these partitions by gratings, and thence, after coming into contact with a considerable surface of pipe, is admitted through several apertures to the interior of the cells." The chimney is said to be capable of drawing 1,059,300 cubic feet of air per hour, which, as there are 1,200 cells altogether, is equivalent to 882·7 cubic feet per cell per hour, or double the quantity required. By means of registers in the air vaults, the ventilation can be made uniform. Our authors commend this system, call it "very admirable", and "worthy of imitation by governmental boards in this country." We congratulate these gentlemen that they speak only of this ventilation from hypothesis; for if they had tried it practically for thirty-six days, as has one who is known to the writer, they would possibly substitute "execrable" for "admirable", and recommend our "governmental boards", in the name of humanity, to try no such experimental tricks on British soil.

Another plan of warming and ventilating is that of M. Duvoir. This has been carried out in the Hôpital de Lariboisière, and in many other public buildings in Paris. In this plan an open reservoir for hot water is placed in a large main shaft at the top of the building. The radiation of heat from this reservoir rarifies the air in the main shaft, into which transverse shafts open, which receive a series of ventilating tubes from all the wards of the building. The ventilating tubes run up the walls of the wards, and have two openings communicating with the wards, one at their upper, the other at their lower part. By closing the upper openings the room can be ventilated from beneath, and all impurities can thus be swept downwards; by closing the lower openings and opening the upper ones, the wards can be ventilated by a current upwards. The external air is freely let into the wards by numerous channels. Duvoir's plan is said to work well, and to be inexpensive.

The ventilation of the Newcastle Infirmary, executed by Mr. Dobson, is for many reasons the simplest and best of all the artificial systems with which we are acquainted. We must trespass again on the authors of the *Chemical Technology* for a description of this scheme.

"The wards are double. They are divided by a wall, in which are the open fireplaces and ventilators. This wall is perforated with large circular openings to allow a free communication for the air from window to window, which can be regulated according to the direction of the wind. When open, the sash of the window is at an angle of inclination, causing the cold air to enter above the heads of the patients. Cold air is also admitted at the table foot at a sufficient distance from the beds to produce no inconvenience. The outside walls are built hollow, having an air vent, three inches wide, communicating with the atmosphere by air holes at the top and bottom. A current of air is thus established, which prevents the deposition of moisture on the walls. From this vent the cold air is conveyed by an air channel along the beams which carry the floor, and is admitted at the table leg, where there is a valve which can be closed at pleasure. The contaminated air is removed from the wards by exhaustion on a simple plan. The fireplaces in the parallel wards are placed back to back, having a malleable iron air chamber between them, protected from the action of the fire by fire clay lining. It is perforated at the top and bottom to allow the atmosphere, which is supplied to it from the room below, to become heated and pass off by the ventilating flue. Thus the heat of the fire in the ward above is made to ventilate the ward below." (p. 248.)

This plan is said to be at once simple and efficient. In the House of Commons, a plan of Dr. Reid's is adopted.

"The air is supplied from Old Palace-yard to the basement of the building; passing first through a filter, 42 feet long by 18 feet 6 inches deep, for the exclusion of visible soot, it arrives at the heating apparatus, consisting of large chambers intersected by steam pipes, and proceeds from thence to other chambers, where it can be mixed with cold air and brought to any required temperature. The floor of the house is double, and the space below the floor can be connected by means of valves with the hot air chamber. The floor is perforated by a great number of apertures, and these are covered with air cloth to diffuse the current. The air having performed its functions ascends to the ceiling, which is also double and perforated, whence it is carried off by the draught created by a powerful fire up a chimney shaft erected in another part of the building." (*Chemical Technology*, p. 251.)

The House of Lords is ventilated on a simpler plan; which is the less remarkable, because one more complicated could not possibly have been conceived.

"The floors of the rooms are simply heated by the passage of hot air beneath them. The hot air then escapes by passages along the external sides of the rooms to the ceiling, which is divided into two compartments, the one for the admission of the warm air entering at the sides, and the other for the exit of the vitiated air. The warm air, after passing below the floor to the roof, becomes somewhat cooled, so that its temperature on entering the ceiling is a few degrees lower than that actually present in the room; it consequently descends to the level, at which it is at once heated again, and deteriorated by respiration, it rises through the centre of the room through the ceiling to a foul air chamber above, whence it is conducted to a chimney. In the chimney it is carried off by a motive power first suggested by Dr. T. Richardson. This power consists of a jet of steam, which when produced under pressure of 32 lbs. to the square inch, is capable of setting 217 times its bulk of air in motion : 10,000 cubic feet of air are thus gradually diffused per minute, no draught is perceptible, and no inconvenience from dust." (*Chem. Tech.*, p. 251.)

The exhaustion process through a shaft, by means of heat, has been applied in various other ways. In mines, a fire in the ventilating shaft acts exceedingly well. The upper manufactory rooms of Mr. Goode, of Birmingham, are ventilated, as was pointed out to us during a late visit to this establishment, by the simple plan of placing a refrigerator gas burner within a cone from a shaft ascending through the roof of the building. The air is admitted freely from without by the doors and other openings in the walls, and the draught through the shaft is most effective. This arrangement, adapted to use by Mr. Goode, junior, answers better than any previous one which he has tried.

The ventilation of gas burners is a subject that has received great attention. We have already said that the principle of the so-called "syphon ventilator" is good for this purpose, for we have seen it applied in this manner, and we find that it causes a brisk current of air to pass downwards through the burner into the chimney, and not only removes the products due to the combustion of the gas, but assists also to some extent in ventilating the room in which the burner is fixed.

A few years since, Mr. Faraday invented a ventilating gas burner. This consists of an Argand burner with a glass chimney, both of which are enclosed in an outer and larger glass cylinder, which is closed at the top by a plate of mica. The plate at bottom on which this outer cylinder and the lamp chimney rest, has an opening between the outer and inner glass cylinders, from which runs a pipe ending in a ventilating flue. The air to supply the flame is supplied by a separate pipe. When the lamp is in action, the air passing up the chimney lamp cannot escape at the top into the room, being prevented by the mica plate; it descends in the space between the two glass cylinders, and escapes by the ventilating flue. The great disadvantage of this plan is that the current of hot air passing between the two glass cylinders renders the glass opaque. Mr. Rutter has invented a mode of meeting this objection, but as we are of opinion that a tube from the gas burner into a chimney on Dr. Chowne's suggestion is without any other addition sufficient for the end desired, it is not necessary to enter into further descriptions. In a room furnished with a well acting Arnott valve, the evils of the gas burner are also mainly removed.

The second method of ventilation is by a pumping or other mechanical exhausting process. We have referred to plans of this kind in speaking of Dr. Hales' invention. For modern improvements in this direction, the public are much indebted to Dr. Arnott, whose single ventilating pump admits of wide application. His gasometer ventilating process is also exceedingly ingenious, and is used in the York Hospital. It is, in a sentence, an air-pump. An air cylinder or gasometer moves up and down in a case more than twice as deep as itself, within which in its middle part is formed as a lining to it a thin circular trough of water, into which the open mouth of the cylinder plunges as it works. The case has at top and bottom certain valves on each side opening in opposite ways, and as the cylinder plays up and down in the case, pure air is alternately received into and driven out of the spaces below and above the cylinder, so that whether the cylinder be rising or falling, air is being pumped onwards into the ventilating channels of the house. In the York Hospital, the machine is worked by a small water engine; eight complete strokes of the pump are made per minute, giving a ventilation of 2,000 cubic feet in the same time.

A further plan of warming and ventilating at the same time is also the invention of Dr. Arnott, under the title of the " double current warming ventilation". In this process, by means of the ventilating pump, the heated impure air which accumulates at the top of a crowded room, is made in passing through tubes to transfer its heat to pure air passing through other tubes from another pump to feed the room.

Other schemes of ventilation by fans, ventilating pumps, steam jets, and the like, have, at various times, been employed. The principle is the same in all; Hales' bellows, improved upon or modified, and his Highness the Prince, to whom we have before introduced the reader, disgusted as usual, and in most cases obstinate. When Dr. McWilliam went on the Niger expedition, the steamers engaged in the expedition were ventilated by Dr. Reid on the " plenum and vacuum principles." A fanner, or ventilating machine, was put in motion either by the machinery of the steam engine, or by the "krooman"; or when, in the river, the paddles being disconnected from the engine, by the paddles themselves, which acted as water wheels. From the ventilator a series of tubes proceeded to all the compartments of the vessel. When the fanner worked on the "*vacuum principle*", the vitiated air was drawn by it from the various compartments, and was discharged at an opening in the circumference of the fan box. When the "*plenum principle*" was resorted to, the fresh external air was connected with the centre, and blown into the distribution tubes to the several compartments. By this means it was hoped that, under any circumstances, fresh air might be infused into, or vitiated air extracted from the hold, or any part of the vessel. At some periods in this voyage the air was driven through a medicator, with the intention of removing carbonic acid, and evolving chlorine. How far this medication was useful, Dr. McWilliam does not "pretend to determine". The ventilation simply seems to have been tolerably successful.

We turn now to the third mode of ventilating, the true natural system, that, namely, of letting the atmosphere take its own course, giving it entrance everywhere, and escape everywhere. It may be that, under some circumstances, this system cannot be fully carried out, as in the well of a ship, and in underground rooms. In such cases the simplest of the artificial plans of ventilation may be useful; and Arnott's valve is generally advantageous in any room where there is a chimney, except in cases where a large ventilating shaft, distinct from the chimney, is also in action when the play of the valve is nullified.

But whenever it is possible to give the atmosphere free and natural vent, the best ventilation is at once procured, while the secret of the avoidance of draughts lies not in shutting out the atmosphere, but in letting it in freely. Men do not take colds when bathed in the atmosphere out of doors; nor would they in their houses if the atmospheric influence were as general within doors as it is without. But when one part of the body is exposed to a warm atmosphere, and another to a cooling draught fram some chink of a window, the circulation through the skin is of necessity rendered unequal, and a cold is the result. The absolute rule of ventilation ought to be, *to admit air into dwellings as freely as light;* a difficult thing to do, it is granted, but the end, after all, to be had in view. On this point Dr. Steele says: "Ventilation may be subdivided into two branches; the theoretical and the practical. It is useless to deny that the majority of these attempts", the artificial or theoretical, "have signally failed", and he then quotes the words of the Newcastle Committee of Inquiry on Ventilation, which run thus: "With regard to ventilation, we have seen a great diversity of systems, and observed that the most complicated and expensive is that which has generally been found to be the least effective. In some we found furnaces and towers, built specially for this purpose, but now entirely thrown aside. In those whose ventilation was most perfect, we found the system most simple and natural."

The ventilation of rooms, whether wards of hospitals or in private houses, should be effected by free openings for both the entrance and the egress of the atmosphere. The doors and windows admit, when properly arranged, for the entrance, the chimney, when properly arranged, for the exit. The windows should extend from the floor to the ceiling of every room, they should open freely, and the plan of introducing some perforated glass panes, as proposed by Dr. Steele, should be universally adopted. Rooms also, whether wards or private rooms, should properly communicate by at least two of their sides with the air, and in isolated houses, by all sides, so that a free air current may pass through, and the force of the wind be brought into play, from whatever quarter it may blow.

As regards the chimney of a room, we believe that the old fashioned open chimney is the best for ventilation, and the open fireplace by far the healthiest for warming. This opinion about open chimneys, as we have seen, was held by Dr. Hales, and we beg the reader not to run away with any idea of discomfort in thinking of a revival of this old practice. Living once for some weeks in a room with a chimney open to the ceiling, we experienced no inconvenience whatever, but on the contrary, found all the pleasure arising from the inhalation of an atmosphere constantly removed from within, and renewed from without. Downward draughts and damps of smoke were unknown, and might be prevented in all cases by care in construction. Certainly the open chimney removes the possibility of the richly laden mantelpiece and the magnificent mirror. But what of that? who wants to admire his own shadow at the expense of breathing his own poison?

The hospital at Bordeaux, described by Mr. Roberton, is ventilated in the simplest way, viz., by having isolated wards, and these open to the air, from side to side, and from end to end, by means of long windows, so that a current is always passing through " in correspondence with the natural laws of the atmosphere".

In carrying out this, the only natural plan of ventilation, the perforated zinc or glass plates are most useful. Through them the air passes finely distributed, to use a common term, and the danger of the " draught" is removed.

A few words yet have to be said regarding the amount of air that each individual requires. A very difficult question is here involved, since we do not always take in the same amount of air, nor always give up the same amount of carbonic acid. Some persons, it is true, have fixed arbitrary rules. It has been said that two hundred and twelve cubic feet of air per hour are required to remove all the exhalations of the body, and that a certain number of cubic feet in space are required to secure good health. But practical facts set the calculators' arguments aside, and show that everything depends on the mode of ventilation. Place a man in a coffin with plenty of holes in it, so that his Highness the Prince aforesaid can stroll through it at pleasure, and the man certainly won't die from want of air. Place the man in St. Paul's; seal him up there for good, so that his Highness the Prince can in no way and nowhere wedge himself in, and the man will die in time from actual want of air. A slave, in a slave-ship, has been made to exist in a space of fourteen cubic feet; in the London Hospital, some of the patients are allowed to luxuriate in a space of one thousand seven hundred cubic feet.

There is, in fact, no strict rule in reference to cubic space; the safest approximation to a rule is this; to give to each person as much space as possible, and to ventilate that space as freely as possible. In hospital wards, under any system of ventilation, one thousand cubic feet should possibly be the minimum allowance. In private houses, especially in the sleeping-rooms, not less than five hundred cubic feet should be secured.

It is not our intention here to dwell on the subject of the effects of impure air on health. The emanations from the bodies of men and animals are chiefly carbonic acid, ammonia, and water. The poisonous character of the first of these when inhaled is well known. Dr. Bence Jones assumes, from the experiments of Le Blanc, that air containing one per cent. of carbonic acid indicates so impure a state of the atmosphere, that if breathed for twelve hours it would prove injurious. In large doses, the acid kills at once, by arresting the respiration and the oxidation process. What the mischiefs are which arise from the inhalation of small quantities of ammonia we have yet to learn.

In conclusion, we have the honour of being entrusted with an important message. His "veritable Highness the Prince of the Power of the Air" commands us to convey to all whom they may concern, These,—1. That his Highness knows what he is about. 2. That, as his duties are onerous and special, he dislikes interference. 3. That all meddlers who try to cripple him in his plans, and to make him act in accordance with their limited knowledge, will find themselves checkmated. 4. That he is taken with Dr. Arnott, and has no objection to the ventilating valve, *pro tem.*, but would urge that gentleman to invent for his countrymen a new kind of house, through which he, the Prince, could walk with fewer obstructions. 5. Lastly, that it is his Highness's pleasing duty to do all in his power—which, without boast, is great—for the good of mankind; that from the performance of this duty he never rests, but that he is continually prevented from accomplishing his most useful intents by the ignorance, the selfishness, and the meddlesomeness of those who depend on him for life itself.

---

